# An investigation of how fungal infection influences drug penetration through onychomycosis patient’s nail plates

**DOI:** 10.1016/j.ejpb.2016.03.008

**Published:** 2016-05

**Authors:** W.J. McAuley, S.A. Jones, M.J. Traynor, S. Guesné, S. Murdan, M.B. Brown

**Affiliations:** a Department of Pharmacy, School of Life and Medical Sciences, University of Hertfordshire, College Lane, Hatfield, Hertfordshire AL10 9AB, UK; b Pharmaceutical Science Division, King’s College London, Franklin-Wilkins Building, 150 Stamford Street, London SE1 9NH, UK; c Department of Pharmaceutics, UCL School of Pharmacy, 29-39 Brunswick Square, London WC1N 1AX, UK; d MedPharm Ltd, Unit 3 Chancellor Court, 50 Occam Road, Surrey Research Park, Guildford GU2 7AB, UK

**Keywords:** Barrier, Fungal, Nail, Onychomycosis, Topical drug delivery

## Abstract

The treatment of onychomycosis remains problematic even though there are several potent antifungal agents available for patient use. The aim of this investigation was to understand whether the structural modifications that arise when a patient’s nail become infected plates influences the permeation of drugs into the nail following topical application. It was hoped that through improving understanding of the nail barrier in the diseased state, the development of more effective topical treatments for onychomycosis could be facilitated. The permeation of three compounds with differing hydrophobicities, caffeine, terbinafine and amorolfine (clog *D* at pH 7.4 of −0.55, 3.72 and 4.49 respectively), was assessed across both healthy and onychomycosis infected, full thickness, human nail plate sections. Transonychial water loss (TOWL) measurements performed on the healthy and diseased nails supported previous observations that the nail behaves like a porous barrier given the lack of correlation between TOWL values with the thicker, diseased nails. The flux of the more hydrophilic caffeine was twofold greater across diseased in comparison with the healthy nails, whilst the hydrophobic molecules terbinafine and amorolfine showed no statistically significant change in their nail penetration rates. Caffeine flux across the nail was found to correlate with the TOWL measurements, though no correlation existed for the more hydrophobic drugs. These data supported the notion that the nail pores, opened up by the infection, facilitated the passage of hydrophilic molecules, whilst the keratin binding of hydrophobic molecules meant that their transport through the nail plate was unchanged. Therefore, in order to exploit the structural changes induced by nail fungal infection it would be beneficial to develop a small molecular weight, hydrophilic antifungal agent, which exhibits low levels of keratin binding.

## Graphical abstract

**Figure:**
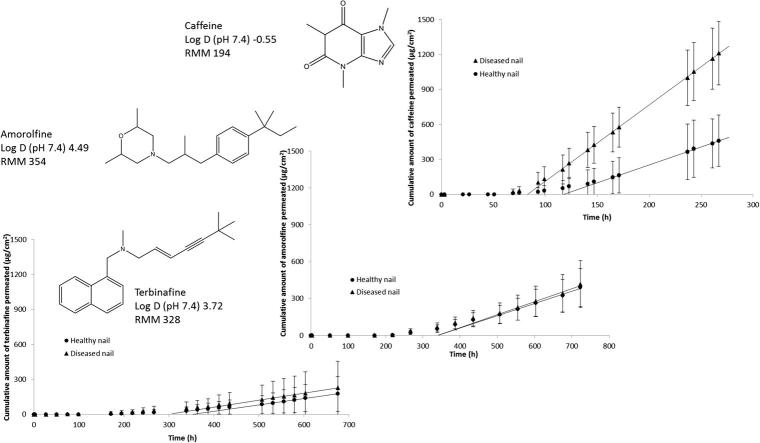

## 1 Introduction

Onychomycosis is a fungal infection of the nail that accounts for approximately 50% of all nail disorders and affects toenails considerably more often than fingernails [1], [2]. The prevalence of onychomycosis has been estimated at around 5% in Western countries and has continued to increase in recent decades [3], [4], [5], [6], [7], [8], [9]. It is important to treat onychomycosis as it is an infection that does not resolve spontaneously, and it may cause pain and can substantially reduce the quality of life of patients [10], [11]. In addition, if left untreated the infection may worsen, spread to other uninfected locations (other nails or to the surrounding skin) or infect other patients. This is especially true for immunocompromised patients and diabetics where the infection can cause major complications [11]. Although, at present, nail removal and oral administration of antifungals are the most effective options for the treatment of onychomycosis, their cost, as well as the toxicity and drug interactions associated with oral drug therapy [12], [13], [14] means that direct application of a therapeutic agent to the nail plate is the most popular approach with clinicians and patients.

A number of topical treatments are currently licensed for onychomycosis and the local application of these products does not carry the risks of serious side effects (such as hepatotoxicity) associated with systemic antifungals and they are less painful than full or partial nail removal. However, all of the topical products require repetitive re-application over long treatment times (e.g. 12 months for toenail infections) [15]. One approach to potentially reduce the treatment time is to enhance drug penetration through the nail plate. The provision of a sufficient amount of the antifungal should resolve the infection as the potency of the drugs used for onychomycosis therapy is excellent, however the biopharmaceutical problem of improving xenobiotic penetration through the nail plate is not easy to address due to the effective barrier properties of the nail [16], [17], [18], [19], [20], [21], [22], [23].

One reason why so little is known about the onychomycotic nail is that there is not a suitable pre-clinical animal model that can be used to validate new formulations prior to their use in patients. This has led to the majority of formulation development and nail characterisation being performed *in vitro* or ex vivo using healthy human nails [24], [25], [26]. The availability of diffusion chambers that can hold very small cross sections of nail tissue has now made it possible to try and address the dearth of information on the structure and properties of fungally-infected nail plates. These chambers have recently demonstrated that onychomycotic nail plate infection obtained in vivo, which increased the nail plate thickness and porosity and reduced its tensile strength and density without disrupting disulphide bonding and/or desmosomes, increased nail permeability when an aqueous vehicle was applied to the nails apical surface [27]. The conclusion that the onychomycotic nail was more permeable than healthy human nail tissue was in agreement with the only other published study in this field [28], but more data were required to understand whether this increase in nail permeability could be exploited by traditional antifungal agents which are normally more hydrophobic than the molecules used in the preliminary work. Hence, the aim of this study was to further investigate the permeability of infected nails using an ex vivo nail model that employed onychomycosis infected patient’s nail using two anti-onychomycotic drugs with differing physicochemical properties (terbinafine and amorolfine; log *D* pH 7.4 values of 3.72 and 4.49 and molecular weights of 328 and 354 respectively) and compare this with a hydrophilic model drug caffeine (log *D* pH 7.4 of −0.55, molecular weight 194). It was anticipated that by challenging the nail barrier with these three molecules in order to understand how the differing barrier function of diseased nail plate affect the ungual absorption, new strategies to improve the topical treatment of onychomycosis could be developed. To achieve this the ChubTur™ cell was used to determine drug penetration into the nail [26], [27], [29] and the nail barrier was characterised using TOWL.

## 2 Materials and methods

### 2.1 Materials

Calibrated ChubTur® diffusion cells were kindly loaned by MedPharm Ltd (Guildford, UK). Tetrahydrofuran was purchased from Acros Organic (Geel, Belgium). Terbinafine HCl and Amorolfine HCl were provided by TCI (Oxford, UK) and Caffeine by Sigma–Aldrich (Dorset, UK). Fisher Scientific (Leicester, UK) supplied triethylamine, orthophosphoric acid, acetonitrile, absolute ethanol, sulphuric acid, propan-2-ol, glacial acetic acid and sodium acetate. Phosphate buffer saline tablets were purchased from Oxoid (Hampshire, UK).

### 2.2 Nail clippings: collection and storage

Healthy nail clippings were collected from volunteers (REC/B/10/01 School of Pharmacy Ethics Committee, University of London). Diseased nail samples were supplied by podiatrists and dermatologists (ethics approval, PHAEC/09-24, School of Pharmacy Ethics Committee of the School of Pharmacy, University of Hertfordshire and ethics approval, 12/YH/0381, NRES Committee Yorkshire & The Humber – Sheffield). The procedures employed for the storage of healthy and diseased nail clippings were developed with a view to retaining the nail in its natural state prior to analysis. For healthy nail clippings, removal of all dirt and debris and washing with water prior to storage were adopted to remove any surface contaminants that could influence the analytical tests. These two stages are associated with everyday cleaning of nails in vivo and therefore cleaning by removal of debris and washing with water were not expected to introduce any significant change in the healthy samples. Healthy nails were kept in sealed polyethylene bags at room temperature prior to use. Diseased nails were kept in sealed vials at 4 °C as this temperature does not affect fungi viability [30] but it inhibits its growth. Nail dimensions were taken using a micrometer (RS components, Corby, UK) and were conducted at room temperature at ambient relative humidity (40–60%).

### 2.3 Transonychial water loss measurement (TOWL)

Nail pieces were trimmed to approximately 3 × 3 mm and the width, breadth and thickness of the nail samples were measured with a micrometre as aforesaid. The nail sections were then clamped and held in place between the receiver and donor chambers of calibrated ChubTur® diffusion cells, as described previously [26]. The dorsal area exposed to the donor chamber was 0.0314 cm^2^. The receiver chambers of each cell were filled with deionised water and a stirring bar was introduced. The receiver fluid of each cell was briefly degassed by sonication and the cells were inverted to remove the resulting air bubble from the ventral face of the nail clipping. The nail clippings were initially left in the calibrated ChubTur® diffusion cells for 24 h to allow the nail samples to hydrate and reach steady state. The condenser-chamber AquaFlux AF200 (Biox Systems Ltd., London, UK) was then used to measure transonychial water loss (TOWL). Each recorded TOWL measurement was determined over a 5 min period and if this showed a typical and reproducible water vapour density curves over this time the cells were used for the permeation studies. In contrast, any setup that showed an atypical water vapour density curves such as no transient peak or extensively long equilibration times to the steady state TOWL value was rejected. After successful completion of the TOWL measurement, each cell was emptied and left to dry prior to conducting drug permeation studies.

### 2.4 Permeation studies

Both healthy and diseased nails were mounted in ChubTur™ diffusion cells which were used for the permeation studies. The receiver chambers of each cell were filled with relevant receiver fluid and a stirring bar was introduced. For caffeine the receiver fluid was PBS pH 7.2, and for terbinafine and amorolfine it was PBS pH 7.2/Ethanol (1:1, v/v). These fluids were chosen to ensure adequate solubility of the drug and that sink conditions were always maintained in the receiver fluid throughout the permeation experiments. For comparative purposes, approximately 44% and 20% of terbinafine (p*K*a 7.1) and amorolfine (p*K*a 6.6) would be expected to be ionised respectively in an aqueous environment at pH 7.2. In contrast caffeine (p*K*a 0.6) would be expected to be unionised. The stability of each drug in the receiver fluid was also confirmed (data not shown). As before with the TOWL experiments, the receiver fluid of each cell was briefly degassed by sonication and the cells were inverted to remove the resulting air bubble from the ventral face of the nail clipping. The receiver chamber was then sealed with Parafilm® and the receiver fluid of diffusion cells was then stirred and acclimatised overnight in a water bath maintained at 37 °C, which provided a nail surface temperature of 32 °C. Saturated solutions of caffeine, terbinafine and amorolfine were prepared by incubating and stirring an excess of solid for 24 h in their respective receiver fluid. The saturated solutions were filtered, and then applied to the dry clean donor chamber (ca. 5 mL) so that the level of the penetrant solution aligned with that of the side arm of the receiver chamber. The donor chambers were then sealed with Parafilm® for the duration of the experiment to prevent evaporation of donor solutions after which the receiver fluid was sampled at predetermined time points. At each time point and before sampling, the cells were visually inspected and inverted 3 times. Replicate experiments were performed for each drug with *n* = 5–7 for the caffeine experiments and *n* = 11–13 and 13–15 for the amorolfine and terbinafine experiments respectively.

### 2.5 HPLC analysis

The amount of caffeine, terbinafine and amorolfine in the samples was quantified by a dual pump Agilent Technologies Infinity 1260 high performance liquid chromatography (HPLC) system (Agilent, Stockport UK) equipped with an autosampler and connected to a UV/Vis detector. This system was connected to a PC with Agilent ChemStation software for data acquisition and collection. The HPLC methods used for each drug were adapted from those kindly provided by MedPharm Ltd (Guildford, UK). For the quantification of caffeine an Agilent Zorbax Eclipse Plus C18 HPLC column, 150 × 4.60 mm 3.5 μm was used in conjunction with an Agilent Reliance Guard column hardware kit containing an Agilent ZORBAX Reliance C18 4.6 × 22.5 mm cartridge. The injection volume was 10 μL. An isocratic method was used with a mobile phase composition of sodium acetate buffer (pH 4.5)/acetonitrile/tetrahydrofuran 95.5/2.5/2 (v/v/v). The flow rate of the mobile phase was 1 mL/min, the column temperature was 23 °C and the UV detection wavelength was 275 nm. The retention time of caffeine under these conditions was 8.9 min.

For terbinafine quantification a Phenomenex® Luna C18(2) 100A 150 × 2.00 mm 5 μm HPLC column (Phenomenex, Macclesfield, UK) was used in conjunction with a Phenomenex Security Guard Column holder containing a C18 4 × 2 mm cartridge. The injection volume was 10 μL. Again an isocratic method was used with a mobile phase composition of orthophosphoric acid and triethylamine buffer (pH 2.5)/Acetonitrile 60/40 (v/v). The flow rate of the mobile phase was 0.3 mL/min, the column temperature was 23C and the UV detection wavelength was 224 nm. The retention time of terbinafine under these conditions was 7.1 min. The same specification of HPLC and guard columns as was used for the analysis of terbinafine concentrations was used to quantify amorolfine. Again an isocratic method was used, however the mobile phase composition was adjusted to 55/45 (v/v) orthophosphoric acid and triethylamine buffer (pH 2.5)/acetonitrile. The flow rate of the mobile phase was 0.3 mL/min, the column temperature was 23 °C and the UV detection wavelength was 219 nm. The HPLC methods were validated for linearity, precision and accuracy according to the current ICH guidelines with correlation coefficient (*R*^2^) values greater than 0.999, accuracy values of 100 ± 2% and relative standard deviation values (RSD) of less than 2% for both repeatability and precision measurements for all three drugs [31], [32].

### 2.6 Data analysis

The cumulative amounts of drug penetrating the nail per unit surface area (μg/cm^2^) were corrected for previous sample removal and plotted against time (*h*). The slope of the linear portion of the permeation profile (*R*^2^ > 0.95) was estimated as the pseudo steady-state flux (Js) of penetrant permeation. The lag times (*L*) for each drug were derived from the *x*-intercept of the slope at pseudo steady-state and used to provide as estimate of the diffusion coefficients (*D*) of the drugs, with *D* being calculated as *D* = *h*^2^/6*L* where *h* is the nail thickness. The permeability coefficients (Kp) of each drug were calculated as drug flux/drug concentration in the donor chamber. The results were expressed as mean ± standard deviation (SD) and were statistically analysed with IBM SPSS software. Data were tested to determine whether they were normally distributed or not and then analysed using either a *t*-test or nonparametric Mann–Whitney *U* test for parametric and nonparametric data respectively. For multiple comparisons, ANOVA was used with post hoc analysis performed with Tukey’s test. Pearson correlation was used to examine the relationship between parameters measured in the study. Statistical significance was accepted at the *p* ⩽ 0.05 level.

## 3 Resu**l**ts and discussion

### 3.1 Nail permeation

**Fig. 1:**
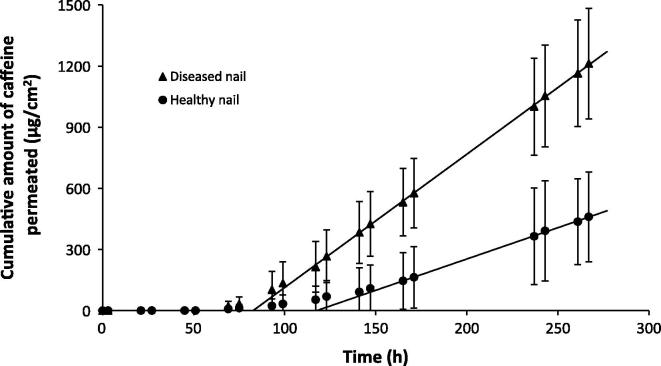
Permeation profiles of caffeine across healthy (●) and diseased (▴) nail samples. Data are shown as the mean ± SD.

**Fig. 2:**
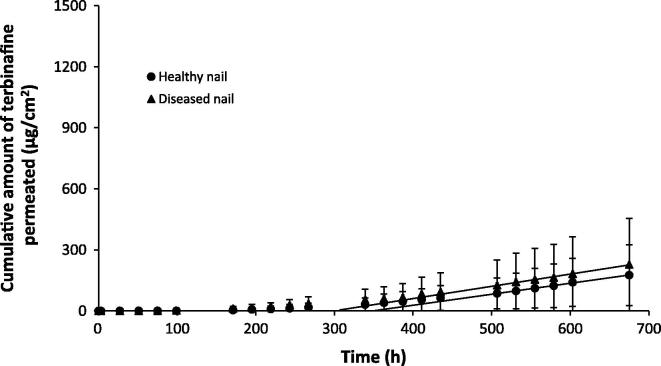
Permeation profiles of terbinafine across healthy (●) and diseased (▴) nail samples. Data are shown as the mean ± SD.

**Fig. 3:**
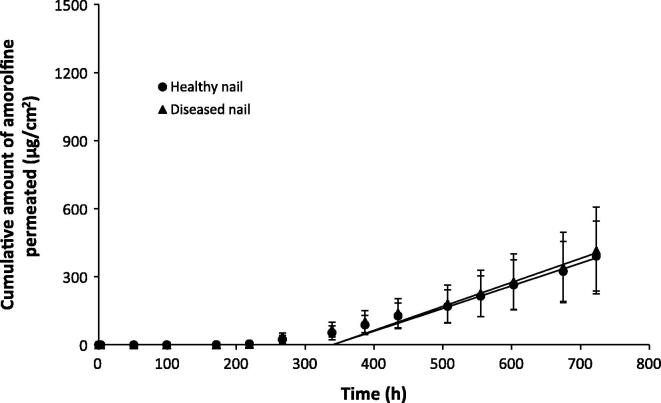
Permeation profiles of amorolfine across healthy (●) and diseased (▴) nail samples. Data are shown as the mean ± SD.

The permeation profiles of caffeine, terbinafine and amorolfine across health and diseased nails are shown in Fig. 1, Fig. 2, Fig. 3 respectively. The lengthy lag period prior to a linear increase in the cumulative amount of drug permeated in these infinite dose experiments is typical of nail permeation experiments [33]. The length of the lag time makes analysis of the data using Fick’s first law of diffusion difficult as steady state flux should be measured from approximately 2.7 times past the lag time [34]. Nonetheless the linear portions of the graph can be used to provide estimates of, or ‘pseudo’ steady state flux and allow estimation of lag times. Table 1 displays the permeation parameters calculated for each of the drugs across healthy and diseased nails along with thickness of the nails used in the experiments.

The diseased nail plates, sourced from the patient volunteers, were thicker than their healthy equivalents (Table 1). Onychomycosis increases the nail plate thickness when the disease is acquired in vivo [27]; therefore, this difference in nail tissue thicknesses across the two different patient groups was considered unavoidable. As a consequence the subsequent results are discussed with relation to this disparity in the diffusion barrier thickness where it was felt important to the study’s conclusions. The study’s data interpretation was not further complicated by any discrepancy across the thicknesses of the tissues employed for each of the three drugs beyond that which has already been mentioned, i.e. both the health and diseased groups of nails used for the caffeine, terbinafine and amorolfine experiments were not statistically different across the three drug types (ANOVA, *p* > 0.05).

The disease state had a large influence of the permeation profiles and parameters of caffeine, with the *in vitro* flux, lag time, diffusion coefficient and maximum quantity of caffeine permeating through the nail all being significantly different for healthy and diseased nails (*p* < 0.05, *t*-test) (Fig. 1 and Table 1). The mean values for the flux, diffusion and permeability coefficients and the maximum amount of drug that permeated through diseased nails were more than double the corresponding values for the healthy nails, whilst the lag time was approximately 1.5 times shorter. In contrast to caffeine, terbinafine and amorolfine nail permeation showed negligible differences between healthy and diseased nails (Fig. 2, Fig. 3 and Table 1) for the drug flux, permeability coefficient and total quantity of drug permeated. When the data were normalised to account for the greater thickness of the diseased nails, differences in the diffusion co-efficient were obtained, with higher values being obtained for diseased nails (*t* test, *p* < 0.05). However as the changes in nail anatomy are inherent to the disease, the permeation data conclusions were drawn from the data which were not corrected in terms of nail thickness, as this was thought to be a better representation of the challenges faced by real products in vivo. The presence of ethanol in the receiver fluid and donor suspension for both terbinafine and amorolfine was necessary given the low aqueous solubility of both drugs. However its inclusion was not thought likely to alter the nature of the differences between healthy and diseased nails and so the comparison of the data with that of caffeine where ethanol was not present was considered valid for comparison of permeation across healthy and diseased samples.

When the three permeant molecules were compared with one another, drug flux was highest and lag time was the shortest for caffeine through both healthy and diseased nail plates (ANOVA, post hoc Tukey, *p* < 0.05) whilst there were no statistical differences between the respective values for terbinafine and amorolfine (ANOVA, post hoc Tukey, *p* > 0.05). Obtaining the highest ungual flux with the smallest, most hydrophilic molecule, caffeine (MW 194), through both healthy and diseased nails correlates with the notion that the human nail plate can be considered as a porous barrier which is amenable to penetration by hydrophilic xenobiotics [35], [36]. Previous studies have shown that it is highly problematic to isolate the effects of the two important physicochemical properties that influence nail penetration, i.e. molecular weight and hydrophobicity, and the data presented in this study do not attempt to make any claims regarding the relative importance of these molecular characteristics; this has been discussed by previously published studies [28], [37]. Rather, it appears more appropriate to use the data from the current study to support the previously purported notion that the nail is a hydrophilic gel membrane, which favours the permeation of water-soluble compounds [20], [37]. This is because the caffeine (MW 194, calculated log *D* of −0.55) nail penetration, like fluorouracil (MW 130; log *D* at pH 4 of −0.96) [28], [38], penetrated through the diseased nail barrier, which has a greater porosity [27], more rapidly, whilst the two hydrophobic antifungal drugs showed little change in permeation parameters. This is in agreement with results obtained by Nair et al. who found terbinafine permeation across onychomycosis infected human nail to not be significantly different to previously published work across healthy nail tissue [39]. Overall this type of behaviour is typical of gels that swell in response to hydration to a certain point, influencing their penetrability by polar molecules, but not by hydrophobic agents that have a tendency to adsorb the gel. This may explain why the effects of permeant lipophilicity on nail permeation are not so well understood. When previous investigations have used an homologous series of compounds, e.g., n-alkanols and p-hydroxybenzoic esters to understand the effect of lipophilicity on nail permeation, they have assumed that the nail is a highly confluent barrier like the skin [22], [37]. This theoretical basis seemed to drive the previous suggestions of Walters and coworkers that there was a lipidic pathway through the nail plate which allowed a greater permeation of decanol and dodecanol, compared to lower alcohols (C3–C8) [22], [23]. However, more recent work, which showed no enhancement of nailplate permeability to terbinafine upon defatting (incubation in chloroform: methanol 2:1 mixture for 12 h), implied that there was something incorrect in the assumption that the nail is a highly confluent barrier [40]. The current study strongly suggests that it is not the tight packing of cells in a strong lipid matrix that provides the barrier properties of the tissue, rather it was the highly tortuous porous route which provided a challenge for penetration. Using this paradigm to understand the data it is easier to incorporate the fact that keratin binding to the nail is known to influence nail penetration. Amorolfine and terbinafine have been reported to bind strongly to keratin [41]. This type of keratin binding would be unlikely to change significantly when pores were opened by the disease and hence it would be unlikely that the disease would change the passage of the hydrophobic molecules.

### 3.2 Nail barrier properties

**Fig. 4:**
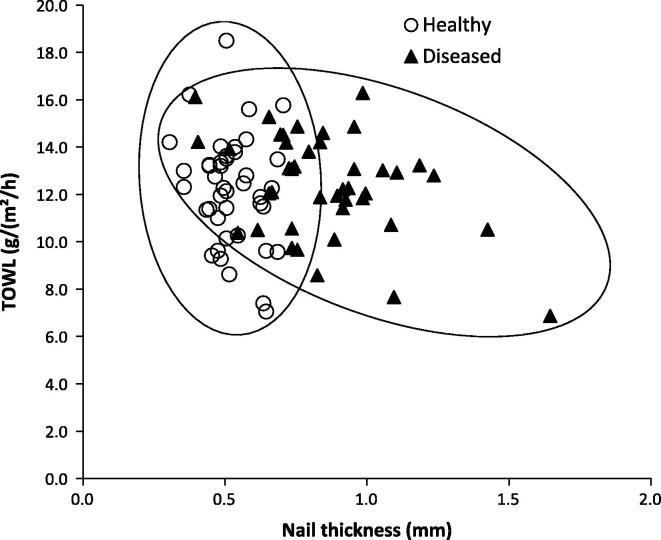
TOWL plotted against thickness for both healthy and diseased nails.

The diseased state had no influence on TOWL (Table 1), i.e., no significant difference was found (*t*-test, *p* > 0.05,) between the TOWL measurements of healthy and diseased nails. This was a little surprising as the diseased nails were known to be more porous and hence one would expect a great amount of water to be lost from the nail. When the TOWL values were plotted against thickness for each nail sample there were two clusters of points corresponding to healthy and diseased nails (Fig. 4), however there was no discernible correlation between the parameters with the separate clusters observable relating to the increased thickness of diseased nails. The similar TOWL values of the (thicker) diseased nails and (thinner) healthy ones could be a result of the greater porosity of diseased nails which allows a free movement of water vapour. The TOWL data interpretation noted here matches the previous reports that have measured TOWL and tissue thickness across a number of patient volunteers. For example, Jemec et al. showed no correlation between these parameters across 21 individuals [42]. Other previous work, using a gravimetric measure of water loss, showed that the nail thickness increase seemed to compensate for the greater porosity when the infection was acquired in vivo and this normalised the water loss across the diseased and healthy nails [27]. Combining the TOWL data in this study and the water loss data from the previous work again supported the notion that the nail thickening was an adaptive change by the body in response to the infection in order to maintain the local tissue’s water homeostasis. The previous work that demonstrated that this nail thickening did not occur when the infection was induced *in vitro* again supports this adaptation hypothesis [27].

### 3.3 Penetration route

**Fig. 5:**
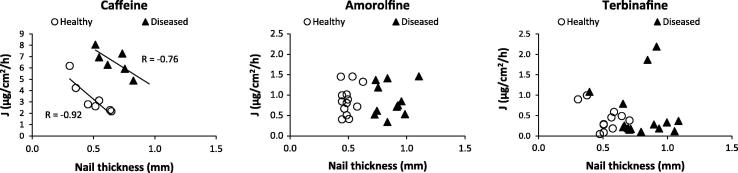
Drug fluxes from the permeation data for caffeine, amorolfine and terbinafine across healthy and diseased nails plotted against nail thickness.

**Fig. 6:**
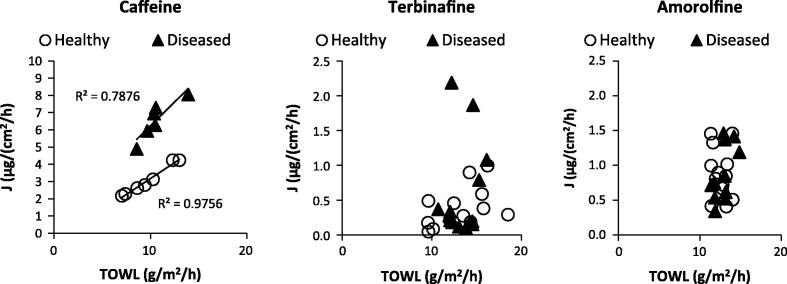
Drug fluxes from the permeation data for caffeine, terbinafine and amorolfine across healthy and diseased nails plotted against nail TOWL value.

The nail plate thickness was expected to influence ungual permeation and it influenced to some extent the pathlength of tissue through which the molecules had to travel in order to pass the nail, and so this was explored further by plotting drug flux for each nail sample against its thickness and the Pearson correlation coefficients (*r* values) were calculated (Fig. 5). An influence of nail thickness on permeant flux was only observed for caffeine with *r* being −0.92 and −0.76 for healthy and diseased nails respectively, with the correlation being statistically significant (*p* < 0.05) for the healthy nails only. The lack of any kind of correlation for the terbinafine and amorolfine (*p* > 0.05) data supported the notion that keratin binding plays a greater role in their permeation across nail rather than the nail acting as a simple diffusional barrier. When the drug flux was plotted against TOWL for each nail, a significant correlation (*p* < 0.05) was observed for caffeine permeation, with *r* values of 0.99 and 0.89 for healthy and diseased nails respectively with no correlation observable for terbinafine or amorolfine (Fig. 6). The strength of this correlation was thought to be an indication that the hydrophilic caffeine passed through healthy and diseased nails via the same diffusional pathway as water and that TOWL may provide useful insight into the nail barrier to the permeation of small hydrophilic molecules. In contrast it does not appear to be useful in explaining the nature of the nail barrier to hydrophobic molecules. Moreover as TOWL did not vary with disease state despite the structural changes that this produces, it is suggested that TOWL cannot be used as an indication of nail plate integrity and the barrier in general, in contrast to the equivalent transepidermal water loss (TEWL) which is often used as a measure of skin integrity and barrier function. This again demonstrates that the skin and the nail act as very different barriers. The characteristics of an onychomycosis infected nail may differ in vivo in comparison with when it is used in an ex vivo experimental design, for example as a result of differences in nail hydration. As such care should be taken in directly applying these findings to the clinical situation. In addition it would be beneficial to confirm these findings with a larger sample size and assessment of a wider range of molecules. However the lack of an appropriate preclinical in vivo model for ungual permeation means that currently, the ex vivo use of healthy, and the more difficult to obtain diseased nail tissue, is considered to be the most suitable model for investigating drug permeation across the nail and understanding the nature of the nail barrier [43].

## 4 Conclusion

This report appears to be the first published study to systematically investigate the ungual permeation of anti-onychomycotic agents through diseased nails donated by patient volunteers. This was made possible using the specialised ChubTur diffusion cell which is capable of holding a small sample of the diseased nail plate obtained from patient volunteers during an ex vivo permeation experiment. Whilst ungual permeation of the small hydrophilic molecule caffeine was greatly increased in diseased nails there was no change in the fluxes and lag times of the hydrophobic antifungals terbinafine and amorolfine. Thus the commonly held assumption that diseased nail is more permeable than its healthy equivalent appears only to be true when applying a small and hydrophilic molecule to the nail surface. This is unfortunate when it is considered that most drugs marketed for the topical treatment of onychomycosis are lipophilic in nature and thus it is not surprising that such products have long treatment times. Although an effort is being made in developing new chemical entities (e.g. tavaborole Mwt. 152.0 and cLog *P* 1.24) with better properties for ungual penetration, this study suggests that the focus for the future should be on drugs that are even more hydrophilic so that they can take advantage of the decreased barrier of the diseased nail.

## Figures and Tables

**Table 1:** TOWL and thickness of the healthy and diseased nails used in the permeation experiments along with the drug flux, diffusion coefficient and total drug quantity permeated for caffeine, terbinafine and amorolfine. Data are shown as the mean ± SD.

Drug	Nail condition	Nail thickness (mm)	TOWL prior to experiment (g/m^2^/h)	Drug flux (μg/cm^2^/h)	Lag time (h)	Diffusion coefficient (×10^−4^ mm^2^/h)	Permeability coefficient (×10^−5^ cm/h)	Total drug quantity permeated (μg/cm^2^)
Caffeine	Healthy	0.5 ± 0.1	9.7 ± 2.3	3.07 ± 0.86	121.5 ± 33.2	3.5 ± 0.9	17.6 ± 4.9	460.3 ± 220.3
Diseased	0.7 ± 0.1	10.6 ± 1.8	6.56 ± 1.11	84.3 ± 16.1	8.8 ± 1.8	37.7 ± 6.4	1211.8 ± 271.0

Terbinafine	Healthy	0.5 ± 0.1	13.3 ± 3.0	0.41 ± 0.30	339.8 ± 115.3	1.6 ± 0.9	7.1 ± 5.2	149.8 ± 123.0
Diseased	0.8 ± 0.2	13.3 ± 1.5	0.55 ± 0.66	327.6 ± 98.2	3.7 ± 2.0	9.5 ± 11.4	228.1 ± 311.3

Amorolfine	Healthy	0.5 ± 0.1	12.6 ± 1.0	0.89 ± 0.36	299.8 ± 37.1	1.4 ± 0.4	1.6 ± 0.6	390.9 ± 154.3
Diseased	0.9 ± 0.1	12.8 ± 1.1	0.89 ± 0.40	284.3 ± 14.6	4.5 ± 1.3	1.6 ± 0.7	416.0 ± 190.8
